# Reflections on the withdrawal of medical articles and the development of writing and publishing standards

**DOI:** 10.20892/j.issn.2095-3941.2022.0437

**Published:** 2022-09-23

**Authors:** Lidong Wang, Zhihua Liu

**Affiliations:** 1 Research Integrity Construction Office (RICO), National Natural Science Foundation of China, Beijing 100085, China; 2 The Supervisory Committee, National Natural Science Foundation of China, Beijing 100085, China; 3 State Key Laboratory of Molecular Oncology, National Cancer Center/National Clinical Research Center for Cancer/Cancer Hospital, Chinese Academy of Medical Sciences and Peking Union Medical College, Beijing 100021, China

In recent years, the withdrawal of several batches of articles from international academic journals has negatively affected Chinese scholars. The Chinese government, the scientific community, and many scientific researchers have made substantial efforts to rectify this problem. This Editorial reviews the mass withdrawal events as a whole; identifies their causes; and systematically investigates China’s policy adjustments, institutional arrangements, and regulatory mechanisms in response. We hope that, in addition to reminding authors to pay greater attention to avoiding withdrawals, this Editorial will provide guidance to help them conduct scientific research and present their achievements in a more standardized manner.

## Overview of batch withdrawal

The mass withdrawal from international journals occurred in 2015. On March 26, 2015, BioMed Central revoked 43 articles, 41 of which were by Chinese authors, because of forged peer reviews. On August 18, 2015, Springer announced that it had revoked 64 articles published in 10 of its academic journals, some by Chinese authors, because of forged peer reviews. On October 13, 2015, Elsevier revoked 9 articles in 5 of its journals, all by Chinese scholars, because of forged peer reviews. On December 18, 2015, Nature Publishing Group revoked 3 Chinese scholars’ articles because of forged peer reviews. The above batches of withdrawals prompted great concern among the Chinese government, competent authorities and the public. Among the withdrawn articles, 28 were either supported by or used to apply for National Natural Science Foundation of China (NSFC) projects. The NSFC performed a special investigation of the above articles and responded in accordance with the relevant laws and regulations.

The mass withdrawal of *Tumor Biology* articles in 2017 brought the issue of the scientific integrity of research articles to the forefront of public opinion. On April 21, 2017, the Springer group announced that it would withdraw 107 articles published in *Tumor Biology*, one of its journals, from 2012 to 2016. All articles were by Chinese authors, including 524 physicians from well-known hospitals and medical schools in China. The Ministry of Science and Technology of China verified that among the 107 articles withdrawn, 2 articles had been repeatedly published in *Tumor Biology*; 1 article had been withdrawn by mistake, the authors were not at fault, and *Tumor Biology* issued a public clarification; 101 articles had provided false peer review experts or false peer review opinions, of which 95 involved false peer review experts or false peer review opinions provided by third-party institutions, and 6 involved false peer review experts or false peer review opinions provided by the authors themselves. Of the 101 articles, 12 had been purchased from third parties, and the remaining 89 had been completed by the authors. After academic review, 9 were found to have false content, and the other 80 articles were found to have been completed by the authors and to not contain false content. Statistical studies have shown that China contributed 8.2% of the scientific research articles published worldwide (journal articles indexed by SCI/SSCI/AHCI) but 24.2% of the withdrawn articles from 1978 to 2017^1^.

Since then, reports of mass withdrawals from international journals have occurred every year to date. The *American Journal of Cellular Biochemistry*, in a special supplement in October 2021, separately published 129 withdrawn articles by Chinese scholars. Of course, this practice is questionable.

Researchers have comprehensively analyzed the reasons for the withdrawal of articles and divided them into 39 categories^2^. However, according to the authors’ experience, the reasons for the withdrawal of articles in batches since 2015 have changed from traditional and common plagiarism and repeated publication to the concealed manipulation of peer review and the use of technical means to write articles on behalf of others. Commercial third-party companies have played a key role, gradually converting the former manual workshops into “factories” for the mass-production of articles. In cases involving third-party companies, the boundary between normal scientific research task entrustment and inappropriate commercial operation does not appear to be very clear.

## Reasons for batch withdrawal

The causes of the problems leading to withdrawal events have been summarized by researchers. The corresponding authors involved in withdrawals have mainly been clinicians. In addition to their time-consuming clinical work, they must publish SCI articles to meet the criteria for their performance appraisals, professional title reviews, and project reviews. However, owing to time limitations, a lack of experience, insufficient English-language skills, and other issues, they may fall into the trap of hiring a third-party company^3^.

We have previously studied the causes of research misconduct^4^, focusing on writing and publishing articles, and have identified the following main reasons.

1. Quantitative error tendency
  Since there is no scientific and reasonable judgment standard currently, it is difficult to evaluate the scientific level of scholars from different fields, or even the same field. The scientific article is widely recognized as a standard carrier of intellectual achievement. As a result, an inappropriate tendency to treat the number of articles and the quality of articles as a positive correlation has gradually formed due to its implementation cost and simplicity, and it has slowly spread to many fields and even become a recognized rule.
2. Unreasonable evaluation mechanism
  The evaluation mechanism is unreasonable. Under normal circumstances, students must publish articles at a certain level to obtain a degree, and researchers must publish articles to gain professional title promotions or scientific funding. However, in reality, researchers are often judged according to evaluation criteria guided by theory, regardless of whether students receive academic-oriented or skill-oriented training; whether teachers specialize in education and teaching or in educational and scientific research; or whether physicians are full-time clinicians or engage in teaching, clinical practice, and scientific research.
3. Improper interest linkage
  The aforementioned misdirection of over-emphasis on articles further leads to over-rewards for publishing articles. The quantity and quality of articles, in addition to providing an important reference index for professional advancement, are also closely associated with the incomes of the authors of articles. The excessive academic awards and scientific research performance awards given in the past become an important source of income for researchers.
4. Limited means of Academic Misconduct Technology Detection Information technology is limited; electronic means of information storage and comparison have not been fully realized; and technical tools such as duplicate checking have only recently been popularized. Even if the accuracy of technical detection methods can be greatly improved, due to the fact that the database has not been fully shared, it is difficult for editors to detect potential academic misconduct in submitted articles in a timely manner.
5. Low cost of breaching academic norms
  In the past, China did not pay sufficient attention to publicizing and popularizing academic norms and scientific research rules, and educating researchers; thus, many researchers have low awareness of research integrity and rules of conduct. For various reasons, these violations could not be addressed in the past^5^. However, this situation has since changed considerably.

## Regulatory policy to prevent batch withdrawal

To effectively curb misconduct in scientific research, nurture the spirit of science and of scientists, abide by ethical guidelines, and build a healthy academic ecology, the general office of the Communist Party of China (CPC) Central Committee and the general office of the State Council formulated and issued programmatic documents on scientific research integrity, the spirit of scientists, and scientific research ethics in 2018, 2019, and 2022. The 3 documents are closely linked and complement one another, providing a normative, favorable, high-quality sustainable development approach with a top-level design.

In May 2018, the general office of the CPC Central Committee and the general office of the State Council issued several opinions on further strengthening scientific research integrity, which defined the guiding ideology, basic principles, and main objectives and tasks of ensuring scientific research integrity in the new era. These opinions provide an important institutional basis for further strengthening scientific research integrity in China in the new era. They highlight the principles of no restricted areas, full coverage and zero tolerance for all academic misconduct, including investigating and addressing acts in violation of scientific research integrity, as well as lifelong accountability for such acts, in accordance with laws and regulations. Various enterprises, institutions, and social organizations that engage in scientific research are the first parties responsible for ensuring scientific research integrity, and the unit to which the violators belong is the first party responsible for the investigation and response.

In June 2019, the general office of the CPC Central Committee and the general office of the State Council issued opinions on further advancing the spirit of scientists and strengthening the good academic and research behavior.

These documents describe personality requirements for most scientific and technological workers at the emotional level; encourage and guide most scientific and technological workers to pursue truth; establish the concepts of value that are widely recognized by the scientific and technological community; and promote the normalization and institutionalization of style of study. China will strive to fully implement the various governance measures within 1 year and achieve substantial changes in the style of study within 3 years.

In March 2022, the general office of the CPC Central Committee and the general office of the State Council issued opinions on strengthening the governance of science and technology ethics, requiring that science and technology ethical requirements be applied throughout the entire process of scientific research, technological development, and other scientific and technological activities, to achieve responsible innovation. Units engaged in scientific and technological activities, such as in the life sciences and medicine, with research content involving sensitive aspects of scientific and technological ethics, should establish a scientific and technological ethics committee, and the people in charge of scientific research projects should perform research in strict accordance with the scope approved by the scientific and technological ethics review board. Scientific and technological ethics norms and guidelines should be formulated in key areas such as medicine. Colleges and universities, scientific research institutions, and medical and health institutions are the first parties responsible for the internal investigation and management of scientific and technological ethics violations.

In accordance with the requirements of the above 3 policies, to unify the rules for the investigation and management of scientific research integrity cases, the Ministry of Science and Technology led 20 national institutions in jointly formulating and issuing these rules. Additionally, 41 national institutions signed a memorandum of cooperation regarding the joint discipline of dishonest actors in the scientific research field, and 38 national institutions signed a memorandum of cooperation regarding the joint discipline of severely dishonest actors in the field of intellectual property (patents), thus laying an institutional foundation for establishing and improving a joint mechanism for discipline in response to dishonesty in the field of scientific research.

The laws on scientific and technological progress were established in 1993 and revised for the first time in 2007, which was the second major revision after 14 years. The revised law on scientific and technological progress was officially implemented on January 1, 2022. The revision focuses on improving measures for safeguarding scientific and technological innovation, improving the national innovation system, and striving to remove obstacles to independent innovation, providing a legal guarantee for the promotion of self-reliance and self-improvement in high-level science and technology.

In addition, relevant professional management organizations have formulated regulations, systems, industry standards, blue books, and other normative documents within the industry to provide specific operation guide.

For example, the NSFC has formulated measures for the investigation and treatment of research misconduct in NSFC projects; the National Health Commission has revised and released a code of integrity and related conduct for medical research; and the National Press and Publication Administration has officially released China’s first industry standard for academic misconduct, academic publishing codes and definitions of academic misconduct in journals (cy/t 174-2019). The Institute of Scientific and Technical Information of China and others have also released a blue book on the pitfalls of using third-party editing agencies in scholarly publishing.

## Mechanism for addressing batch withdrawal

First, an early-warning mechanism for periodical publications should be established. The Ministry of Science and Technology is responsible for establishing an early warning mechanism for academic journals, carrying out “network clearing action”, supporting relevant institutions in publishing an early-warning list of domestic and international academic journals, and implementing dynamic tracking and timely adjustments. Academic journals whose disregard for academic quality, confusion in management or acting in accordance with commercial interests have led to adverse effects will be blacklisted. The unit to which the authors of the article belong must remind authors who publish articles in the academic journals on the list in a timely manner and not recognize them in reviews, or reimburse them for expenses associated with the publication of the article. Institutions of higher learning, scientific research institutes, and medical institutions have also adjusted and released lists of early-warning periodicals in their fields and closely related fields.

Second, a representative work should be applied. Malicious academic fraud and sales of SCI articles have occurred, thus further demonstrating that the standard of simply using publication quantity to evaluate the quality of scientific research work is not scientific or rational. Therefore, a strong movement has emerged in support of dismantling the system of “only articles, only professional titles, only academic qualifications, and only awards”. The party and the government have proposed a classified evaluation system based on the quality, contributions, and performance of scientific and technological innovation, which highlights morality, ability, and performance; the quality, contribution, and impact of the achievements; the implementation of a representative work evaluation system; and avoidance of emphasizing quantity over quality or a “one size fits all” approach. The NSFC has resolutely implemented a representative work system and clearly requires that representative achievements be limited to 5 items in applications for project funding. Shandong Province has clearly required that the number of representative works for major basic research projects, outstanding youth funding projects, and major soft science research projects must not exceed 5 in principle, and the number of representative works for other types of projects must not exceed 3 in principle. Since 2019, Tsinghua University has no longer required postgraduates to publish SCI articles. On April 29, 2022, the Department of Education of Jiangxi Province and the Department of Human Resources and Social Security of Jiangxi Province jointly issued a document that college teachers in Jiangxi Province can apply for the title of professor as long as they have one treatise or one high-quality article.

Third, joint discipline is conducted under the social credit system. In November 2018, 41 national institutions signed a memorandum of cooperation regarding the joint discipline of dishonest actors in the scientific research field; consequently the relevant initial information on misconduct in the scientific research field will be used in the social credit system, and offending researchers may face restrictions in jobs, loans, and business opportunities far beyond their academic careers^6^.

Fourth, legal regulation should be instituted. As the core vehicle of achievement in scientific research, articles not only showcase completed or exploratory research but also are used to obtain continuing funding or to support new applications for funding. After problems occur, they affect not only the reliability of past research results but also the availability of follow-up funding. In today’s society, articles and projects involve many practical or potential legal issues, such as the crime of fraud, the crime of falsifying documents, the crime of intentional injury or homicide in criminal law, and tort and compensation liability in intellectual property law and other civil laws.

Foreign countries have long had systems to legally regulate articles and other achievements involving scientific research misconduct and related funded projects. In 2005, Eric Poehlman, an American scholar, was sentenced to US $180,000 in civil liability and 1 year and 1 day in prison under US law for systematically tampering with key data supporting his aging theory, and forging and tampering with data in a project funding application. In 2009, South Korean scholar Hwang Woo Suk was charged with fraud, misappropriation of research funds, and illegal trading of eggs. Subsequently, he was sentenced to 2 years’ imprisonment with a 3-year suspension by the Seoul District Court.

The Chinese government also clearly requires that behaviors that severely violate the requirements of scientific research integrity be subject to lifelong accountability, and actively performs theoretical research on the criminal regulation of such behaviors. On December 30, 2019, the case of the “gene editing baby” was first publicly announced in the Shenzhen Nanshan District People’s court. Jiankui He and 3 other defendants were investigated for criminal responsibility under the law for their joint illegal implementation of human embryo gene editing and reproductive medical activities for the purpose of reproduction, which constituted the crime of illegal medical practice. The court sentenced defendant Jiankui He to a fixed-term imprisonment of 3 years and a fine of 3 million yuan. In recent years, several judicial precedents have been set involving corruption, embezzlement, fraud, fraudulent claims and misappropriation of scientific research funds. Chinese researchers and research integrity management practitioners have also systematically demonstrated the necessity of a criminal law governing the sale and writing of articles. The revised law on scientific and technological progress, which came into effect in 2022, stipulates that those who engage in trading, writing, and ghostwriting for academic articles and the associated experimental research data, as well as applications and final reports for science and technology projects, will be given a warning or a notice of criticism and fined by the relevant competent departments. The illegal gains, if any, will be confiscated, and if the circumstances are serious, the offender’s license will be revoked.

Good practices in writing, submitting, publishing, and using articles

1. Original data retention and submission for archiving
  The Chinese government and relevant administrative agencies clearly require that original data, such as test records and experimental data, be submitted to the appropriate unit for unified management and retention for future reference within 1 month after the publication of scientific research achievements, such as articles. This process ensures the protection of the original intellectual property rights of Chinese researchers, as well as a reasonable and effective response in cases of dispute.
2. Prohibition on purchasing data
  In recent years, some authors of articles have not performed the experiments themselves but have used the experimental results of other institutions or laboratories to write their articles. In this case, even if the data themselves are correct, the authenticity and reliability of the article are extremely questionable^7^.
3. Boundaries for commissioned tests and review of feedback data
  According to the management regulations of the industry and the department, in combination with relevant industry standards, and with reference to the blue book on the pitfalls of using third-party editing agencies in scholarly publishing, medical researchers can perform some tests in strict accordance with the specifications but must pay attention to the supervision of the testing operation and the review of the feedback data from third-party to ensure the normalization of the commissioned tests, and the authenticity and reliability of the results.
4. Avoiding duplication, partial duplication, split publication, and other low-level forms of publication
  Publishing a certain research achievement in different journals or dividing it into several parts for publication is intended to achieve the highest possible number of published articles.
5. Avoiding plagiarism of the words or thoughts of others
  In writing articles, authors should not copy the written expression of others, or directly use the thoughts, ideas, and schemes of others as their own intellectual output.
6. Representative work system
  Since the reform, the evaluation framework has increasingly focused on the quality assessment of research results in the form of articles, i.e., on the substantive contributions and achievement levels of articles rather than the number of articles. Authors should also pay attention to the relevance of representative works and the declaration of subsequent projects, awards, professional titles, etc.
7. Avoiding improper practices such as accepting gifts and nominal signatures
  In publishing articles, medical researchers should abide by the “Five Prohibitions” for publishing academic articles and the relevant provisions regarding the submission of academic articles and the publication of works. Articles and other achievements should be signed and sorted according to the actual contributions to scientific research achievements, and those who do not make substantial academic contributions should not be included as authors.
8. Careful selection and organization of images
  The first principle should be to strictly abide by scientific principles and authenticity. Authors should select a picture corresponding to the research content with good effect from the real original pictures. The images should be treated in strict accordance with the relevant industry regulations and the editorial requirements of the contributing journals. Artificially assembling and cropping images and research results is strictly prohibited, as is concealing artificial manipulations, such as using different display proportions, or performing horizontal or vertical rotation, or flipping operations.
9. Prohibition on writing or submission of articles by a third party
  Writing and submitting articles with the help of a third party for any reason is prohibited.
10. No false peer review
  Peer review is a classic means of academic evaluation and is important in ensuring academic quality. In submitting contributions, authors must recommend reviewers to journals according to their professional relevance and their academic reputation as relevant scholars. Generating false evaluations of contributions by falsifying the contact information of recommended experts disrupts academic order and wastes many academic resources. It may also mislead readers and cause further waste or damage. In addition, many articles that have been withdrawn due to false peer review were actually completed by a third-party company.
11. Avoiding intentional improper indication of project funding
  Many scientific research projects are supported by funds from various sources. Articles reporting research achievements must disclose any support from a funding project. Project funding support increases the likelihood of acceptance of manuscripts or theses for publication. Consequently, some authors have falsely indicated projects as funded or even misappropriated other researchers’ funded projects. This practice is expressly prohibited.
12. Accurate description of authors’ contributions
  Many journals require that each author’s contribution to an article be explained. This practice is not only a sign of respect for the authors of the article but also a clear indicator of responsibility in the event of future disputes or doubts, and therefore is recommended. Although not every journal will request the information, authorial teams should be encouraged to take the initiative to explain the authors’ contributions.
13. Reference specifications
  Comparison of any reference to academic thoughts or research results are normal academic activities. However, references to other people’s thoughts and conclusions must be clearly indicated; otherwise, readers will be confused about the real owner of the research results. The standard practice is to add a citation where material is referenced so that readers can clearly determine what content is an author’s own and what is a reference from others. It is inappropriate for authors to include the research results of others in the bibliography at the end of an article without citing the specific references in the article.
14. Compliance with ethical approval requirements
  Before performing medical research, researchers must apply for ethical approval in accordance with the regulations. After approval, specific research must be performed in strict accordance with the approved scope and methods in the research process. Relevant ethical approval information should also be indicated in the article.
15. Responsibilities of the corresponding author/tutor/subject leader
  In cooperative research, many researchers normally complete scientific research activities through division of labor and cooperation. However, the roles or responsibilities of each author differ. The corresponding author should take overall responsibility for the design, writing, and quality of the article. Tutors of authors should provide guidance, education, training, and supervision for students. Authors who are project leaders should also check the relevance of the article to the project and the reimbursement of the cost of publishing the article. Relevant state departments clearly stipulate that if students or team members commit misconduct in scientific research activities, the tutors and scientific research project leaders who agree to participate as contributors bear the same responsibility as any individuals directly responsible for the scientific research misconduct, in addition to their leadership and guidance responsibilities.
  Typical cases of scientific misconduct in violation of the above requirements can be easily browsed and queried on the official websites of the relevant national departments, such as the Ministry of Science and Technology, the Ministry of Education, the National Health Commission of the People’s Republic of China, and the National Natural Science Foundation of China.
16. Do not use software to “torture phrases”
  The latest survey has indicated a potential new means of misconduct called “torture phrases”, which results from the use of automated translation or software that rewrites existing text^8^.
